# Knowledge and home treatment of measles infection by caregivers of children under five in a low-income urban community, Nigeria

**DOI:** 10.4102/phcfm.v11i1.1744

**Published:** 2019-04-17

**Authors:** Obioma Uchendu, Olusimbo Ige, Oluwapelumi Adeyera

**Affiliations:** 1 Department of Community Medicine, University College Hospital, Ibadan, Nigeria; 2 Department of Community Medicine, University of Ibadan, Ibadan, Nigeria

**Keywords:** measles, caregivers, home treatment, measles complication, under-5 children, knowledge of measles, measles immunisation, symptoms and signs of measles

## Abstract

**Background:**

Despite the availability of a safe and effective vaccine for over 50 years, measles remains a leading cause of death among young children in developing countries.

**Aim:**

This study assessed the knowledge and home treatment of measles by caregivers of children under 5 years.

**Setting:**

Abebi community, Ibadan, Oyo State, Nigeria.

**Methods:**

A descriptive cross-sectional study of 509 caregivers of children aged 6 months to 5 years in a semi-urban community in Ibadan was conducted using a multi-stage sampling method. An interviewer administered structured questionnaire was used to collect information on socio-demographic characteristics, knowledge of aetiology, main symptoms and signs, and home treatment of measles. Chi-square test and logistic regression were used to explore associations at 5% level of significance.

**Results:**

Most of the caregivers were females (96.3%), married (86.1%) and were the biological parents of the children (90.9%). More than half had good knowledge of the cause (59.7%) and main symptoms and signs (52.8%) of measles. However, the composite knowledge was good in 57.6% of caregivers. Over half (54.4%) of the caregivers reported that their children ever had measles. Majority (91.3%) of caregivers whose children had measles gave home treatment, while 24 (8.7%) sought treatment from health facilities alone. There was a significant association between caregivers’ educational status, age, tribe and marital status and their knowledge of measles; however, tribe was the only significant predictor of knowledge after regression analysis. Caregivers from other tribes were 3.3 times more likely to have good knowledge of measles than Yoruba caregivers. Caregivers who were 35 years and older compared to those younger than 35 years (OR: 0.625; 95% CI: 0.425–0.921) and those who were not currently married compared to those married (OR: 0.455; 95% CI: 0.273–0.758) had lower odds of having good knowledge of measles, respectively.

**Conclusion:**

Home treatment by caregivers of children with measles is high. Health education on the cause, prevention and treatment of measles should be provided for caregivers.

## Introduction

Measles also known as rubeola is an acute, highly communicable viral exanthematous disease caused by a virus.^1^ Measles, a vaccine-preventable disease, is one of the leading causes of death among children.^2^ In 2015, there were about 134 200 deaths globally from measles, translating to about 367 deaths every day or 15 deaths every hour, and during 2000–2015, measles vaccination prevented about 20.3 million deaths.^2^ This implies that vaccination is actually effective against measles infection.^3^ Despite the availability of a safe and effective vaccine for over 50 years, measles remains a leading cause of death among young children in developing countries, especially among non-immunised children.^4^ Efforts have been made for mass vaccination against measles, especially for children; however, in sub-Saharan Africa, measles still ranks high among the burden of vaccine-preventable diseases.^5^

Measles has also been rated as the fifth leading cause of childhood mortality.^2^ The mortality rates of measles are generally low (3% – 5%); however, during an epidemic, it could rise up to 10% of the cases.^5^ Children do not die directly from measles but from its complications when not properly managed.^6^ These complications include ear infection (otitis media), pneumonia, diarrhoea, visual impairment (keratitis and blindness) and subacute sclerosing panencephalitis (SSPE).^1,3^ Children under the age of 5 years or adults over the age of 20 years are usually more susceptible to complications of measles.^2,6^

Factors that predispose to burden of measles morbidity and mortality include poor immunisation coverage, malnutrition, cultural habits that influence health-seeking behaviour and early age of occurrence of infection.^2,4,5^ Other predisposing factors especially in the developing nations are political instability, cultural and religious objections to immunisation^7^ and cultural habits that influence health-seeking behaviour.^8^ Home treatment of diseases such as diarrhoea, malaria, measles and other febrile illnesses has been documented to be frequently practiced by caregivers of children under 5 years. While this practice has been useful in reducing mortality, when poorly instituted by caregivers it worsens disease outcome.^7,8,9,10,11,12^ The importance of proper home treatment as a means of reducing child mortality from febrile illness resulted in World Health Organization (WHO) recommendations to health-educate mothers on home treatment of certain diseases.^13^ As such, the health and well-being of these children depend on proper diagnosis and treatment of illnesses by the caregivers.

Home management of diseases is influenced by caregivers’ knowledge of the aetiology, symptoms and signs including prevention and treatment of the disease.^10,11,12^ However, the social, economic, educational and cultural characteristics of the caregiver determine their knowledge.^8^ One of the five components in the strategy for measles elimination and eradication is early and appropriate case management of measles, which includes treatment given at home especially for uncomplicated measles.^9^ It is therefore important to study the knowledge of caregivers on measles infection and how cases of measles are treated at home. This will aid in instituting appropriate measures to encourage prompt, timely and effective case management and health care seeking behaviour among caregivers. This study therefore examined the knowledge and home management of measles infection by caregivers of children aged 6–60 months and the factors affecting home management.

## Methods

### Study design

This study was a community-based cross-sectional survey.

### Study area

The study was carried out in Abebi, a community located in Ibadan Northwest Local Government Area (LGA), Oyo State. Oyo State is one of the 36 states of Nigeria and is located in the southwestern region of the country. The population of the state is about 5.5 million.^14^ Ibadan is the capital of Oyo State, with a population of over 3 million according to the 2006 census.^14^ Ibadan municipality has five LGAs, namely Ibadan North, North-East, North-West, South-East and South-West. Each of the LGAs is further subdivided into wards.

Abebi, the site of the study, is located in Ward 6 of Ibadan Northwest LGA. The population of the community is estimated to be 14 871 persons.^14^ It is a high-density population area that is grossly unplanned. It is a connecting route to the major commercial centres, namely Oja Oba and Ekotedo markets, Dugbe and Mokola. The community is made up of 68 compounds and 627 houses. There is no formal traditional ruling structure but an extended family system where members of the same family live in the same compound with the family head known as ‘Mogaji’. Most of the inhabitants are of Yoruba ethnicity, low socio-economic status and are Muslims. The major occupation of women in the community is small businesses, while most men are artisans. The community has access to a health centre where antenatal care, routine immunisation services and treatment of common conditions are provided by the Department of Community Medicine, University College Hospital, Ibadan. There are also private clinics and maternities in the area.

### Study population and sampling

This study was a community-based cross-sectional survey of caregivers of children aged 6–60 months residing in selected households. Caregivers were either mothers or any other relative who was a major care provider and was able to give details of the child’s immunisation and health history. A minimum sample size of 455 caregivers was estimated using Leslie Fischer’s formula for sample size calculation with a population of 14 871, a 95% confidence interval, a margin of error of 4.5% and a 50% prevalence of home treatment for measles.

Multi-stage sampling was used to select eligible caregivers. In the first stage, Ibadan North-East LGA was selected from the five LGAs in Ibadan municipality by balloting. The second stage was the selection of Ward 6 (Alawo or Abebi Ward) from the 11 wards in the LGA by balloting. In the third stage, one settlement Abebi was selected from the 21 in Ward 6 by balloting. All the 68 compounds and 627 houses in Abebi settlement were visited. In each house, one eligible household was selected by balloting if there was more than one household in the house. The final stage was the selection of the index child where a household had more than one child between ages 6 and 60 months. The use of all 627 houses was predicated on the assumption that there are approximately four to five households in each house and that there would be a household with a child under 5 years of age.

### Data collection

A structured interviewer administered questionnaire was used to obtain information on socio-demographic characteristics of the index child and caregiver, knowledge of cause, signs and symptoms of measles, immunisation history and type of treatment given.

The questionnaire was adapted by the authors from extensive literature review^15,16,17^ and anecdotal information on cultural beliefs, perceived aetiology, symptoms and treatment of measles infection. The modification by the authors on the questionnaire was based on anecdotal information obtained from caregivers of children in a different locations from the study LGA. Such information included ‘spiritual attack’, ‘mosquito bites’, ‘exposure to heat’, etc, as wrong perceptions on the cause of measles (see Appendix 1). The general wrong perception on causes includes: ‘spiritual attack’, ‘mosquito bites’, ‘exposure to heat’ and so on (see the supplement-questionnaire). The questionnaire was also critiqued and corrected by academics who were involved in measles surveillance. The tool was translated to Yoruba and back-translated to ensure that the original meaning of the questions was retained before being pre-tested in a community outside the chosen LGA. Ambiguous items in the questionnaire were then corrected after the pre-test. Construct validity and reliability of the instrument was, however, not done. Six research assistants who were community health workers were recruited and trained as interviewers for this study.

### Data management

The primary outcome for this study was the use of home treatment for under-five year old children with measles by caregivers, while the secondary outcome was caregivers’ knowledge of measles infection. The domains of knowledge assessed were as follows: ‘knowledge of cause of measles’ and ‘knowledge of the signs and symptoms of measles’. The composite knowledge ‘overall knowledge of measles’ was also obtained from the two domains assessed. The responses to the knowledge questions were ‘yes’, ‘no’ and ‘I don’t know’. Every correct response to a question scored 1 point, while every incorrect response or ‘I don’t know’ scored 0 point. ‘Knowledge of cause of measles infection’ was assessed with 14 questions with a maximum obtainable score of 14 points, while ‘knowledge of symptoms and signs of measles’ was assessed with 16 questions with a maximum obtainable score of 16 points. The maximum obtainable score for ‘overall knowledge’ was 30 points. The mean ± SD knowledge scores were computed for ‘knowledge of cause of measles infection’ (8.2 ± 3.9), ‘knowledge of symptoms and signs’ (8.5 ± 2.1) and ‘overall knowledge’ (15.6 ± 4.2). Respondents whose scores were equal to or greater than the mean scores were categorised as having ‘good knowledge’, while those with scores less than the mean score were categorised as having ‘poor knowledge’.

The WHO classification for ‘probable case of measles’ which is ‘any child with fever of 38.3 °C, generalized maculopapular (non-vesicular) rash lasting ≥ 3 days and cough, coryza (runny nose), or conjunctivitis (red eyes)’ was used as the case definition for measles in this study.^18^ A child was therefore identified as having had measles only if the caregiver diagnosed measles infection in tandem with the case definition for ‘probable case of measles’. Caregivers whose children had measles 1 year prior to or within the study period were included in the study.

### Definition of terms

‘Index child’: this was a child less than 5 years old for which information on measles was sought from the caregiver. The index child was selected by balloting only in households where more than one of the children less than 5 years has had measles infection.

‘Home treatment’ for measles was said to have occurred where a caregiver administered any form of remedy (orthodox or unorthodox) to an under-five year old child with measles (probable case definition) for more than 2 days at home without visiting a health facility.

The immunisation status of the children was determined by caregivers’ reports and confirmed with the immunisation cards.

‘Occupational groups’ included the unskilled (e.g. farmers, traders and housewives), semi-skilled (carpenters and welders) and skilled (e.g. teachers, nurses and civil servants) groups.

### Data analysis

For the purpose of analysis, educational status was categorised into ‘below secondary education’(no formal and primary education) and ‘secondary education and above’; marital status into ‘currently married’ and ‘not currently married’, ethnicity into ‘Yoruba’ and ‘others’ and relationship with child into ‘parental relationship’ and ‘other relationships’.

Data obtained were analysed using Statistical Package for the Social Sciences (SPSS) version 22 software package.^19^ Chi-square test and the logistic regression were used for statistical analysis to explore the factors associated with caregivers’ knowledge of measles and the use of home treatment for their children who had measles. Level of significance was set at 5%.

### Ethical considerations

Ethical approval was obtained from Oyo State Ministry of Health Ethical Review Board and informed consent was obtained from the caregivers after providing information about the study and confirming that they fully understood the study objectives. Confidentiality of the respondents was also ensured.

## Results

A total of 520 questionnaires were administered of which 509 were completed and used for the analysis, giving a response rate of 97.9%. Eleven (2.1%) of the respondents did not complete their interviews.

### Socio-demographic characteristics

The socio-demographic characteristics of the caregivers and children are shown in Table 1. The mean age of caregivers was 31.2 ± 7.6, with 363 (71.3%) of them aged less than 35 years. Most (96.3%) were women, 438 (86.1%) were married, 463 (90.9%) were the parents of the children and 299 (58.7%) of them had secondary education. The mean age of the index children was 29.5 ± 13.7 months, with majority of them (119, 23.4%) being in the 15–24 months age group.

**TABLE 1:** Socio-demographic characteristics of caregivers of under-five year old children.

Variable (*N* = 509)	Frequency	Percentage
**Sex**	
Male	19	3.7
Female	490	96.3
**Age of caregiver**	
Less than 35 years	363	71.3
35years and above	146	28.7
**Ethnicity**			
Yoruba	474	93.1
Ibo	25	4.9
Hausa	8	1.6
Others^†^	2	0.4
**Religion**	
Islam	296	58.2
Christianity	213	41.8
**Marital status**				
Single	20	3.9
Married	438	86.1
Divorced	43	8.4
Widowed	8	1.6
**Relationship of caregiver to child**			
Parents	463	90.9
Mother’s sister	14	2.8
Father’s sister	19	3.7
Grandparent	13	2.6
**Education**			
None	49	9.6
Primary	132	25.9
Secondary	299	58.7
Tertiary	29	5.8
**Occupation**		
Unskilled	64	12.6
Semi-skilled	401	78.8
Skilled	44	8.6
**Family type**	
Monogamous	399	78.4
Polygamous	110	21.6

† , Benin & Efik.

### Caregivers’ knowledge of the cause and main symptoms and signs of measles

**FIGURE 1:**
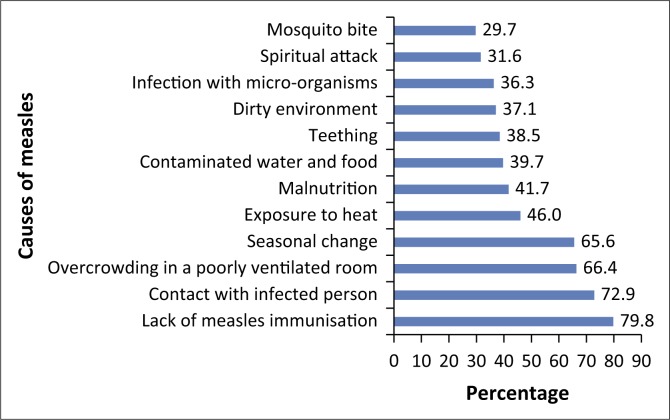
Causes of measles indicated by caregivers (*N* = 509).

All 509 respondents had heard (were aware) of measles infection. Concerning the knowledge of causes of measles, 304 (59.7%) caregivers had good knowledge of the cause of measles (see Table 2). The causes of measles indicated were: lack of measles immunisation (79.8%), contact with an infected person (72.9%), exposure to heat (46.0%) and malnutrition (41.7%) (see Figure 1).

**TABLE 2:** Caregivers’ knowledge of the cause, main symptoms and signs of measles and overall knowledge about measles.

Knowledge (*N* = 509)	Frequency	Percentage
**Knowledge of the cause of measles**		
Poor	205	40.3
Good	304	59.7
**Knowledge of the main symptoms and signs of measles**		
Poor	240	47.2
Good	269	52.8
**Overall knowledge of measles**		
Poor	216	42.4
Good	293	57.6

**FIGURE 2:**
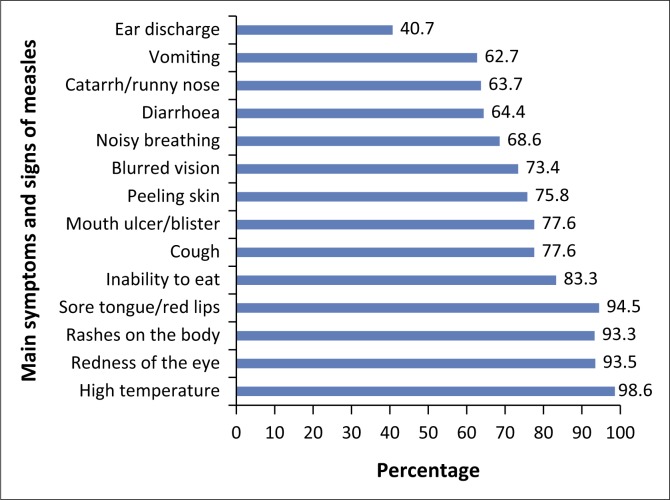
Main symptoms and signs of measles indicated by caregivers (*N* = 509).

Regarding the main symptoms and signs of measles, 269 (52.8%) of the caregivers had good knowledge of the main symptoms and signs of measles (see Table 2). Majority of them correctly indicated that high temperatures (98.6%), redness of eyes (93.5%) and rashes in the body (93.3%) were the main symptoms of measles (see Figure 2).

The overall knowledge of measles expressed by the caregivers (see Table 2) indicated that more than half (57.6%) of them had good knowledge of the disease (i.e. causes, main symptoms and signs of measles).

### Prevalence of measles and immunisation

Probable cases of measles 1 year prior to or during the study period were reported among 277 (54.4%) children less than 5 years of age by their caregivers even though more than three-quarters (387, 76.0%) of the children had received measles immunisation.

### Home treatment of measles

Out of the 277 caregivers whose children had measles, 253 (91.3%) administered home treatment and only 24 (8.7%) opted for treatment from health facilities. Some of the treatments administered at home included palm oil (81.8%), palm wine (77.5), antibiotics (75.1%) and tepid sponge (68.4%) (see Table 3).

**TABLE 3:** Home treatment and remedies used by caregivers of children under the age of five in the home treatment of measles.

Home treatment (*N* = 253)	Frequency	Percentage
**Use of home treatment for measles**		
Yes	253	91.3
No	24	8.7
**Remedies used**^†^		
Paracetamol	216	85.4
Palm oil on the body	207	81.8
Palm wine for bathing	196	77.5
Antibiotics	190	75.1
Tepid sponge	173	68.4
Black soap mixture for bathing	168	66.4
Oral herbs	162	64.0
Drugs for malaria	157	62.1
Bitter leaf for drinking and bathing	157	62.1
Local bin	127	50.2
Cream or lotion	116	45.8
Eye drops	100	39.5
Shea butter mixture	76	30.0
Breast milk in the eye	73	28.9

† , multiple response.

#### Factors associated with caregivers’ knowledge of measles

Caregivers’ educational status, age, tribe and marital status were significantly associated with their knowledge of measles. However, the tribe of the caregivers was the only significant predictor of their knowledge following logistic regression. Caregivers from other tribes were 3.3 times more likely to have good knowledge of measles compared to those of Yoruba tribe (see Table 4).

**TABLE 4:** Association between socio-demographic factors of caregivers and their knowledge of measles.

Variable (*N* = 509)	Knowledge of measles				Unadjusted odds ratio (95% CI)	Adjusted odds ratio (95% CI)
	Poor (*n* = 216)		Good (*n* = 293)			
	*n*	%	*n*	%
**Sex**						
Male^§^	11	57.9	8	42.1	1.912 (0.756–4.836)	1.428 (0.512–3.986)
Female	205	41.8	285	58.2		
**Educational Status**						
Below secondary education^§^	90	49.7	91	50.3	1.586 (1.099–2.288)^†^	1.344 (0.899–2.008)
Secondary education and above	126	38.4	202	61.6		
**Age of caregiver**						
Below 35 years^§^	142	39.1	221	60.9	0.625 (0.425–0.921)^†^	0.751 (0.486–1.161)
35 years and above	74	50.7	72	49.3		
**Religion**						
Islam^§^	136	45.9	160	54.1	1.413 (0.987–2.024)	1.351 (0.931–1.959)
Christianity	80	37.6	133	62.4		
**Tribe**						
Yoruba^§^	209	44.1	265	55.9	3.155 (1.352–7.365)^†^	3.254 (1.364–7.762)^‡^
Others	7	20	28	80		
**Relationship with child**						
Parent^§^	193	41.7	270	58.3	0.715 (0.390–1.311)	1.148 (0.564–2336)
Other	23	50	23	50		
**Marital status**						
Currently married^§^	174	39.7	264	60.3	0.455 (0.273–0.758)^†^	0.567 (0.318–1.009)
Not currently married	42	59.2	29	40.8		

CI, confidence interval.

† , Statistically significant relationship on bivariate analysis (unadjusted odds ratio).

‡ , Statistically significant relationship on regression analysis (adjusted odds ratio).

§ , Reference category.

#### Factors associated with home treatment of measles

There was no significant association between the socio-demographic characteristics and home treatment of measles by the caregivers. However, a higher proportion of caregivers with poor knowledge of measles (93.2%) compared to those with good knowledge (89.9%) administered home treatment though this association was not statistically significant (see Table 5).

**TABLE 5:** Association between caregivers’ socio-demographic factors and knowledge of measles infection with home treatment of measles.

Variable	Home management of measles				Unadjusted odds ratio (95% CI)
	Yes (*n* = 253)		No (*n* = 24)		
	*n*	%	*n*	%
**Sex**					
Male	12	85.7	2	14.3	0.548 (0.115–2.605)
Female	241	91.6	22	8.4	
**Educational status**					
Below secondary education	108	93.9	7	6.1	1.809 (0.725–4.515)
Secondary education and above	145	89.5	17	10.5	
**Age of caregiver**					
Below 34 years	150	90.9	15	9.1	0.874 (0.368–2.072)
35 years and above	103	92.0	9	8.0	
**Religion**					
Islam	151	90.4	16	9.6	0.74 (0.305–1.794)
Others	102	92.7	8	7.3	
**Tribe**					
Yoruba	228	91.2	22	8.8	0.829 (0.184–3.735)
Others	25	92.6	2	7.4	
**Relationship to child**					
Parent	226	91.5	21	8.5	1.196 (0.335–4.274)
Other	27	90.0	3	10.0	
**Marital status**					
Currently married	207	90.8	21	9.2	0.643 (0.184–2.2.46)
Not currently married	46	93.9	3	6.1	
**Overall knowledge of measles**					
Poor	110	93.2	8	6.8	1.538 (0.635–3.725)
Good	143	89.9	16	10.1	

CI, confidence interval.

## Discussion

This study assessed the knowledge of measles among caregivers of children aged 6–60 months and the practice of home treatment for their children’s measles.

More than half (59.7%) of the caregivers from our study had good knowledge of the cause of measles, with over a third (36.3%) of them knowing that measles was caused by a microorganism. This was in contrast to another study conducted in eastern Nigeria, where almost all respondents were aware of measles but none of them knew the cause of measles.^17^ Despite the good knowledge of caregivers in our study, they wrongly indicated that measles was caused by mosquito bites, spiritual attacks, dirty environment, teething, contaminated food and water, malnutrition, heat, overcrowding and seasonal change. Wrong knowledge of the cause of measles (hot weather and malnutrition)was reported among over 90% of clients attending immunisation clinics in Enugu State in a study by Ossai and Fatiregun.^17^ Also, a community-based study conducted in Northern Nigeria among mothers reported that 26% of mothers with children less than 5 years old wrongly attributed the cause of measles to heat and evil spirits.^20^ The knowledge of measles reported in our study can be attributed to the fact that over three-quarters of the caregivers would have been health-educated on measles infection when they took their children for measles immunisation. Therefore, while the need for immunisation is promoted at child welfare and immunisation clinics, there is a need to teach the right causes of measles and demystify the wrong perceptions of the caregivers.

Concerning the knowledge of caregivers about main symptoms and signs of measles, over half (52.8%) of the caregivers of our study had good knowledge. However, complications of measles such as sore tongue or red lips (94.5%), blurred vision (73.4%), inability to eat (83.3%), mouth ulcer or blister (77.6%) and ear discharge (40.75) were regarded as main symptoms and signs of measles. This has significant implication on the morbidity and mortality outcome of measles. Classifying complications as signs and symptoms of early infection means that children are treated at home longer than they should and consequently present late for treatment in health facilities. This potentially leads to the disabling complications (visual, auditory, mobility and learning disability) and case fatality associated with measles.

The overall knowledge of measles (57.6%) among caregivers of less than 5-year-old children reported in our study is higher than the knowledge (16.2%) reported among mothers of less than 5-year-old children in Konduga and Auno districts in Northern Nigeria.^20^ It is, however, lower than the knowledge of measles (87.2%) among mothers of children attending child care centres in Hulu Langat, Selangor, Malaysia.^21^ The high knowledge reported by the Malaysian study can be attributed to their study design. The study was facility-based and was not only on measles alone but also other vaccine-preventable diseases. The studies from Nigeria were both community-based and assessed knowledge of measles infection alone. Furthermore, the difference in knowledge of measles in our study compared to the study by Ambe et al.^20^ in Northern Nigeria can be attributed to higher level of education in southwestern Nigeria compared to the Northern Nigeria.

In this study, only 8.7% of the caregivers took their children to the health facility within 48 hours. Conversely, measles was managed at home by 91.3% of the caregivers in this study. In Northern Nigeria, 69% of mothers were reported to have administered home treatment to their under-five year old children.^20^ Another study among mothers in eastern Nigeria also revealed a high prevalence of home management of diseases among caregivers of under-five year old children.^22^ Several remedies have been documented as the first action initiated by caregivers for home treatment of measles.^12,22,23^ While some of the remedies have no harmful or beneficial effect (e.g. bathing with palm wine and bitter leaf water), some have some beneficial effect (drinking of palm wine that contains vitamin A, palm oil and shear butter mixture on the body to moisturise the skin). However, majority of these remedies have harmful effects, ranging from mild to severe. Mild harmful effects include black soap mixture for bathing which causes dryness of skin. Severe harmful effects include drinking bitter leaf which worsens diarrhoea; instilling breast milk, palm oil and urine in the eyes which can cause blindness; and drinking human or cow urine mixture which worsens diarrhoea and causes renal failure. These severe effects have been known to cause or increase the probability of disabilities.^7^

According to the WHO, any case of measles should be reported to a health facility within 48 h as part of control strategy.^6^ This will allow for surveillance of the disease and ensure that exposed children in the community have measles vaccination following confirmation that it is indeed measles virus. Also, another reason to ensure early visit to the health facility is to commence the child on appropriate treatment including vitamin A supplementation which is known to prevent ocular complications from measles infection. Although the same WHO report suggests commencement of treatment at home like use of antipyretics to reduce fever and administering oral rehydration solution with zinc to prevent dehydration from diarrhoea, the WHO clearly advises that children should be taken to health facility even when measles is suspected.^6^

In this study, associations between socio-demographic characteristics and home management of measles were not significant. Olaogun et al. in 2006 also reported non-significant association between educational level and home treatment of febrile illness.^22^ However, this finding is in contrast with other studies conducted in Nigeria and Malaysia where education, occupation, age, tribe and other factors affected home treatment of measles.^12,22,23^ The reason for this difference can be attributed to the study outcome. Our study focused on home management of measles, while other studies focused on febrile illnesses generally.

A review study on eradication of measles using Nigeria as a case study reported the following reasons for the persistent prevalence of measles in Nigeria: cultural factors, health system and governmental factors.^7^ The review implies that measles will continue to infect children and the complications, case fatality and disabilities will continue to increase if the caregivers continue home treatment especially in children who are malnourished or have poor access to quality health care. Measles infection does not cause mortality but poor case identification and incorrect treatment which occurs at home predispose the children to the complications, disability and death associated with measles.

A major limitation of this study was the use of a questionnaire whose construct was not validated. The non-existence of a standardised instrument may be responsible for the varying knowledge reported by other researchers. However, this study attempted to make the instrument better by including wide range of responses that were generally perceived as cause, symptom and treatment of measles infection. Furthermore, a critical review of the questionnaire by academics involved in measles surveillance provided some content validity to the questionnaire.

## Conclusion

Knowledge of measles was good among half of the caregivers; however, there are still some misconceptions about the causes and symptoms and signs. Caregivers also delay in taking their children to health facilities and administer some harmful home remedies. Information on the cause and identification of measles and other vaccine-preventable diseases rather than on only completing the immunisation schedule should be intensified at antenatal, immunisation and postnatal clinics. The need for early presentation at the health facility to prevent complications from diseases should also be intensified through health education.

## Appendix 1: Questionnaire

### Serial No: __________

- Socio-demographic data
  1. What is your age (last birthday in years)? ____________________
  2. Sex
    - Male
    - Female        
  3. What is your religion?
    - Christianity
    - Islam Traditional
    - Others (specify)____________________        
  4. What is your tribe?
    - Yoruba
    - Ibo
    - Hausa        
    - Others (specify) ____________________
  5. What is your highest level of education?
    - None
    - Primary education
    - Secondary education
    - Polytechnic education
    - University education
    - Other post-secondary education (specify) ____________________        
  6. What is your marital status?
    - Single never married
    - Single cohabiting
    - Married
    - Separated
    - Divorced
    - Widow/widower        
  7. What is your occupation?
    - Housewife
    - Farmer
    - Trading
    - Artisan
    - Professional
    - Others (Please specify) ____________________        
  8. Relationship of caregiver with child
    - Parent
    - Mother’s sister
    - Father’s sister
    - Grandparent
    - Foster parents
    - Others (please specify) ____________________        
  9. Family type
    - Monogamous
    - Polygamous        
  10. Monthly income level of caregiver ____________________
  11. - Who owns the house you live in?
      - Personal
      - Rented
      - Family house        
    - What type of house do you live in? (select only one)
      **Table:**
      Housing type	Storey	Bungalow
      i.	Duplex		
      ii.	Flats		
      iii.	Winged apartments		
  12. How many rooms do you occupy in the house? ____________________
  13. How many children do you have? ____________________
    - Male        
    - Female        
  14. Did all your children receive complete immunisation?
    - Yes
    - No        
  15. If No to Q. 14, how many did not receive complete immunisation?
    - Male        
    - Female        
- Knowledge of cause, symptoms and signs, and complications of measles
  **Table:**
  16.	Measles is common among:	Yes	No	I don’t know
  a.	Children less than 9 months			
  b.	Children between 9 months and 5 years			
  c.	Children between 6 and 18 years			
  d.	Adults			
  e.	Elderly			
  f.	Foreigner			
  g.	Others (specify)			
  **Table:**
  17.	What causes measles?	Yes	No	I don’t know
  a.	Exposure to heat			
  b.	Infection with microorganism/virus			
  c.	Spiritual attack			
  d.	Mosquito bite			
  e.	Physical contact with infected person			
  f.	Inhalation of bad air			
  g.	When a child is malnourished			
  h	Contaminated water or food			
  i.	Dirty environment			
  j.	Bushy environment			
  k.	Overcrowding in a poorly ventilated room			
  l.	Lack of immunisation for measles			
  m.	Seasonal change/change in weather			
  n.	When child is teething (tooth eruption)			
  o.	Others (specify)			
  **Table:**
  18.	The main symptoms of measles are	Yes	No	I don’t know
  a.	High body temperature			
  b.	Cough			
  c.	Running nose/catarrh			
  d.	Redness of eyes			
  e.	Rashes on the body			
  f.	Inability to eat			
  g.	Vomiting			
  h	Diarrhoea			
  i.	Mouth ulcer/blister			
  j.	Fast breathing			
  k.	Ear discharge			
  l.	Noisy breathing			
  m.	Peeling skin			
  n.	Sore tongue/red lips			
  o.	Blurred vision			
  p.	Nightmares			
  q.	Others (specify)			
- Prevalence of measles infection and immunisation rates
  19. Has any of your children had measles before?
    - Yes
    - No        
  20. How many of your children have had measles before? _____________________
    - Male        
    - Female        
    **Table:**
    21	How many of your children have had measles in the last	one year?	Male	Female	Total
  22. Did your children with measles receive measles immunisation?
    - Yes
    - No        
  23. If yes to Q25, where did they receive immunisation? ________________________________________
    If yes to Q. 22, is there immunisation card for confirmation?
    - Yes
    - No        
  23. If no to question Q. 22, why did they not receive immunisation?________________________________________
    **Table:**
    25.	How did you know your child has measles?	Yes	No
    a.	Cough		
    b.	Vomiting		
    c.	Fever		
    d.	Redness of eyes		
    e.	Runny nose/catarrh		
    f.	Rashes		
    g.	Mouth ulcer/blister		
    h	Noisy breathing		
    i.	Pain and swelling behind the ear		
    j.	Malnutrition/severe weight loss		
    k.	Convulsion		
    l.	Rapid breathing		
    m.	Swelling of body and or feet		
    n.	Difficulty in breathing		
    o.	Unconscious/lethargic		
    p.	Chest in drawing		
    q.	Others (specify)		
- Management of measles in children by caregivers at home
  26. In your child with measles, did you give any home treatment?
    - Yes
    - No          If no to Q. 26, go to Q. 30.
    **Table:**
    27.	If yes to Q. 29, what did you do?	Yes	No
    a.	I gave oral herbal concoction		
    b.	I tepid sponged		
    c.	I gave paracetamol		
    d.	I gave drugs for malaria		
    e.	I gave palm wine for bathing/drinking		
    f.	I used eye drops		
    g.	I used herbal concoction for bathing		
    h	I used urine mixture in the eye		
    i.	I used body cream/lotion		
    j.	I used palm oil on the body		
    k.	I gave urine mixture for drinking		
    l.	I used palm oil in the eye		
    m.	I gave antibiotics		
    n.	I used breast milk in the eye		
    o.	I used bitter leaf for drinking and bathing		
    p.	I used black soap mixture for bathing		
    q.	I used shea butter mixture on the skin		
    r.	I gave local gin for drinking		
    s.	Others (specify)		
  28. Did you seek further treatment outside your home?
    - Yes
    - No          If no to Q. 28, go to Q. 37.
  29. If yes to Q. 28, for how many days did you treat at home before seeking further treatment outside the home? ____________________
    **Table:**
    30.	What was the reason for seeking help outside the home?	Yes	No
    a.	Mouth ulcers		
    b.	Dehydration		
    c.	Redness of eye with pains		
    d.	Ear discharge		
    e.	Severe fever		
    f.	Inability to eat or drink		
    g.	Noisy breathing		
    h	Pain and swelling behind the ear		
    i.	Malnutrition/severe weight loss		
    j.	Convulsion		
    k.	Rapid breathing		
    l.	Swelling of body and or feet		
    m.	Difficulty with breathing		
    n.	Unconscious/lethargic		
    o.	Chest in drawing		
    p.	Others (specify)		
  31. Where did you first seek for treatment for your child outside after home treatment?
    - Chemist
    - Herbalist
    - Church        
    - Mosque
    - Relatives
    - Friends
    - Hospital
    - Others (specify) ____________________
    **Table:**
    32.	Why did you choose to seek treatment where you did from Q. 31?	Yes	No
    a.	It was not expensive		
    b.	It was the nearest place		
    c.	Workers were more friendly		
    d.	Pain and swelling behind the ear		
    e.	Staff are always available		
    f.	Workers are more sympathetic		
    g.	They counsel and listen to patients		
    h	Drugs are always available		
    i.	It was recommended by a relative (specify)		
    j.	It was recommended by a friend		
    k.	I did not want my child to be injected		
    l.	Others (specify)		
    **Table:**
    33.	At the place you first sought for treatment in Q. 31, what was done?	Yes	No
    a.	Told to use herbs for drinking		
    b.	Told to use herbs for bathing		
    c.	Told to fast and pray for child		
    d.	Told to use palm wine for drinking and bathing		
    e.	Told to use body cream/ointment		
    f.	Told to use eye drops		
    g.	Told to use oral drug		
    h	Told to take rest		
    i.	Others (specify)		
    **If you sought treatment from a hospital in Q. 31, go to Q. 34. If you did not seek for treatment in a hospital, go to Q. 37**.
    **Table:**
    34.	If you first sought treatment in a hospital, what drugs were you given?	Yes	No
    a.	Eye drop		
    b.	Ear drop		
    c.	Cough syrup		
    d.	Antibiotics		
    e.	Skin (calamine ) lotion		
    f.	Paracetamol		
    g.	Vitamin A		
    h	I was not given any medication		
    i.	Others (specify)		
- Outcome of the measles infection
  35. If you sought care in the hospital, was the child admitted?
    - Yes
    - No        
  36. If yes to Q. 35, for how many days was your child on admission? ____________________
    **Table:**
    37.	In your child with measles, which of these problems/symptoms did the child have?	Yes	No
    a.	Dehydration		
    b.	Noisy breathing		
    c.	Ear discharge		
    d.	Convulsion		
    e.	Lethargy		
    f.	Chest in drawing		
    g.	Inability to eat/drink		
    h.	Difficulty in breathing		
    i	Others (specify)		
    **Table:**
    38.	If you did not seek for help in hospital, how did you know that your child was healed from measles infection?	Yes	No
    a.	The skin peeled		
    b.	Fever reduced		
    c.	The child was able to eat		
    d.	Mouth ulcers/blisters were healed		
    e.	Purging/diarrhoea stopped		
    f.	Breathing became normal		
    g.	Eyes were no longer red		
    h.	Ear discharge stopped		
    i.	Others (specify)		
  39. What was the outcome of the treatment given?
    - Alive
    - Dead        

Thank you.

## References

[1] Orenstein WA, Perry RT The clinical significance of measles: A review. J Infect Dis. 2004;189(Suppl. 1):S4–16. 15106083 10.1086/377712

[2] World Health Organisation (WHO) Measles fact sheet [homepage on the Internet]. 2016 [cited 2017 Feb 01]. Available from: http://www.who.int/mediacentre/factsheets/fs286/en/

[3] Campbell H, Andrews N, Brown K, Miller E Review of the effect of measles vaccination on the epidemiology of SSPE. Int J Epidemiol. 2007;36:1334–48. 10.1093/ije/dym207 18037676

[4] WHO/UNICEF Joint statement: Reducing measles mortality in emergencies [homepage on the Internet]. 2004 [cited 2015 Jan 09]. Available from: http://www.whqlibdoc.who.int/hq/2004/WHO_VandB_04.03.pdf

[5] Saleh JA Trends of measles in Nigeria: A systematic review. Sahel Med J. 2016;19:5–11. 10.4103/1118-8561.181887

[6] World Health Organisation (WHO) Measles WHO fact sheet [homepage on the Internet]. 2006 [cited 2006 June 01]. Available from: http://www.who.int/entity/mediacentre/factsheets/en/

[7] Okonko IO, Nkang AO, Udeze AO, et al Global eradication of measles: A highly contagious and vaccine preventable disease-what went wrong in Africa? J Cell Anim Biol. 2009;3(8):119–40.

[8] Mokashi A Management of a child with measles: Health education to villages [homepage on the Internet]. 2005 [cited 2006 June 20]. Available from: www.hetv.org/resources/index.html

[9] World Health Organization Global measles and rubella strategic plan: 2012–2020 [homepage on the Internet]. 2012 [cited 2017 Feb 20]. Available from: http://apps.who.int/iris/bitstream/10665/44855/1/9789241503396_eng.pdf

[10] Orimadegun AE, Ilesanmi KS Mothers’ understanding of childhood malaria and practices in rural communities of Ise-Orun, Nigeria: Implications for malaria control. J Fam Med Prim Care [serial online]. 2015 [cited 2017 Mar 14];4(2):226–31. Available from: http://www.jfmpc.com/article.asp?issn=2249-4863;year=2015;volume=4;issue=2;spage=226;epage=231;aulast=Orimadegun 10.4103/2249-4863.154655 PMC4408706 25949972

[11] Fatungase KO, Amoran OE, Alausa KO The effect of health education intervention on the home management of malaria among the caregivers of children aged under 5 years in Ogun State, Nigeria. Eur J Med Res [serial online]. 2012 [cited 2015 Dec 07];17(1):11 Available from: http://eurjmedres.biomedcentral.com/articles/10.1186/2047-783X-17-11 10.1186/2047-783X-17-11 PMC3462120 22594678

[12] Olaogun A, Ayandiran O, Olasode A, Adebayo A, Omokhodion F Home management of childhood febrile illnesses in a rural community in Nigeria. Aust J Rural Heal. 2005;(13):97–107. 10.1111/j.1440-1854.2005.00661.x 15804333

[13] World Health Organization WHO informal consultation on fever management in peripheral health care settings: A global review of evidence and practice [homepage on the Internet]. 2018 [cited 2018 Mar 27]. Available from: http://www.who.int/malaria/publications/atoz/9789241506489/en/

[14] National Population Commission Federal Republic of Nigeria 2006 Population and Housing Census. Abuja, Nigeria: National Population Commision; 2010.

[15] Singh SN, Srivastava S Traditional biomedical knowledge of Measles, Mumps & Rubella (MMR): A critical review. Int Trends Libr Inf Technol. 2017;4(2):2–30.

[16] Gupta S, Vidya R, Gupta N, Gupte M Factors precipitating outbreaks of measles in district Kangra of North India: A case-control study. Int J Appl Basic Med Res. 2011;1(1):24 10.4103/2229-516X.81976 23776768 PMC3657943

[17] Ossai E, Fatiregun A Urban–Rural disparities on clients knowledge of cause and preventive measures for childhood immunizable diseases in primary health centers of Enugu State, Nigeria. Int J Trop Dis Health. 2016;14(1):1–11. 10.9734/IJTDH/2016/24221

[18] World Health Organisation (WHO) WHO–recommended standards for surveillance of selected vaccine-preventable diseases. The Department of Immunization, Vaccines and Biologicals. Geneva: WHO [homepage on the Internet] 2008 [cited 2017 Feb 26]. Available from: http://apps.who.int/iris/bitstream/10665/68334/1/WHO_V-B_03.01_eng.pdf?ua=1

[19] IBM Corp IBM SPSS Statistics for Windows, Version 22.0. Armonk, NY: IBM Corp; 2013.

[20] Ambe J, Omotara B, Mandu B Perceptions, beliefs and practices of mothers in the sub-urban and rural areas towards measles and measles vaccination in Northern Nigeria. Niger Trop Doct. 2001;31(2):89–90. 10.1177/004947550103100211 11321281

[21] Che Abdullah A, Nor Afiah M, Rosliza A Practice of childhood immunizations among parents and their associated factors in Hulu Langat, Selangor, Malaysia. Int J Public Heal Clin Sci. 2016;3(6):94–104.

[22] Chukwuocha UM, Nwakwuo GC, Emerole C, Dozie INS, Nwuda OEC Prevalent home management techniques and outcome among mothers of febrile children in Eastern Nigeria. J Public Health Epidemiol. 2014;6(3):111–118. 10.5897/JPHE2013.0591

[23] Nwankwo B, Chukwuocha U, Amadi A, et al Treatment seeking behaviour of mothers for febrile children in some rural parts of Imo State Nigeria: Implications for home management of malaria in endemic areas. Int J Trop Med. 2009;4(3):32–135.

